# Methylation of homeobox genes is a frequent and early epigenetic event in breast cancer

**DOI:** 10.1186/bcr2233

**Published:** 2009-02-27

**Authors:** Stella Tommasi, Deborah L Karm, Xiwei Wu, Yun Yen, Gerd P Pfeifer

**Affiliations:** 1 Department of Cancer Biology, Beckman Research Institute of the City of Hope, Duarte, California 91010, USA; 2 Division of Information Sciences, Beckman Research Institute of the City of Hope, Duarte, California 91010, USA; 3 Department of Clinical and Molecular Pharmacology, Beckman Research Institute of the City of Hope, Duarte, California 91010, USA

## Abstract

**Introduction:**

Aberrant methylation of CpG islands is a hallmark of cancer and occurs at an early stage in breast tumorigenesis. However, its impact on tumor development is not fully determined, and its potential as a diagnostic biomarker remains to be validated. Methylation profiling of invasive breast carcinoma has been largely explored. Conversely, very little and sparse information is available on early-stage breast cancer. To gain insight into the epigenetic switches that may promote and/or contribute to the initial neoplastic events during breast carcinogenesis, we have analyzed the DNA methylation profile of ductal carcinoma *in situ*, a premalignant breast lesion with a great potential to progress toward invasive carcinoma.

**Methods:**

We have utilized a comprehensive and sensitive array-based DNA mapping technique, the methylated-CpG island recovery assay, to profile the DNA methylation pattern in ductal carcinoma *in situ*. Differential methylation of CpG islands was compared genome-wide in tumor DNA versus normal DNA utilizing a statistical linear model in the LIMMA software package.

**Results:**

Using this approach, we have identified 108 significant CpG islands that undergo aberrant DNA methylation in ductal carcinoma *in situ *and stage I breast tumors, with methylation frequencies greater than or comparable with those of more advanced invasive carcinoma (50% to 93%). A substantial fraction of these hypermethylated CpG islands (32% of the annotated CpG islands) is associated with several homeobox genes, such as the *TLX1*, *HOXB13*, and *HNF1B *genes. Fifty-three percent of the genes hypermethylated in early-stage breast cancer overlap with known Polycomb targets and include homeobox genes and other developmental transcription factors.

**Conclusions:**

We have identified a series of new potential methylation biomarkers that may help elucidate the underlying mechanisms of breast tumorigenesis. More specifically, our results are suggestive of a critical role of homeobox gene methylation in the insurgence and/or progression of breast cancer.

## Introduction

Breast cancer is a leading cause of cancer-related mortality in women, claiming over 400,000 lives per year worldwide. At the current breast cancer incidence rates, one in eight women is expected to develop the disease in her lifetime [1]. In spite of the high frequency, however, more than 90% of the breast cancer patients will survive if cancer is detected at an early stage and if treatment is begun promptly. Early detection is therefore extremely crucial for successful treatment and favorable prognosis, and emphasizes the need for new screening strategies for prompt intervention.

It is now widely recognized that aberrant epigenetic modifications play a crucial role in altering gene expression and inducing tumor formation [2]. Methylation of CpG-rich islands encompassing gene promoter regions is especially relevant for the silencing of important tumor suppressor genes and accounts for a growing number of diseases, including breast cancer [3-5]. Several genes involved in cell cycle regulation and apoptosis (CCND2, CDKN2A/p16, RASSF1A), DNA damage response (BRCA1), cell adhesion (CDH1) and cell signaling (ER, RARβ 2) have been reported to undergo promoter hypermethylation in breast carcinoma as well as in other tumor types [6-10]. High levels of some hypermethylated genes can be detected very early, in the ductal lavage and nipple aspirates of patients with ductal carcinoma *in situ *(DCIS) (a stage 0 breast cancer) and stage I tumors, with methylation frequencies comparable with those of more advanced, invasive breast cancers [11]. Epigenetic inactivation can also occur, at different levels depending on the gene examined, in benign diseases such as mammary epithelial hyperplasia and intraductal papillomas – but not in disease-free normal breast epithelium, proliferating lactating breast tissue or stromal cells [12]. In some cases, even the normal breast tissue adjacent to the tumor site can display high levels of promoter methylation, indicating that premalignant epigenetic changes have the potential to spread gradually from the tumor epicenter to the surrounding cells or that a field defect exists that promotes tumorigenesis [13,14]. These data altogether support the evidence that methylation-driven gene silencing is a frequent as well as a relatively early event in breast tumorigenesis and can be used as a tag to detect breast cancer lesions at their very first appearance.

Based on this assumption, several groups have attempted, in recent years, to establish multigene DNA methylation profiles for the detection and classification of breast cancer. Their studies were mostly restricted to methylation-specific PCR analysis or to the array-based screening of limited target panels, and thus failed to interrogate the entire 30,000 CpG island repertoire of the genome [12,15-20]. In addition, these studies as well as genome-wide DNA methylation mapping techniques were usually employed to scrutinize invasive and metastatic breast carcinomas (stage II tumors and higher) [21-25]. These screening methods clearly overlook early epigenetic changes that may precede and/or promote invasive growth formation and offer limited diagnostic applications. Given the scattered and inadequate information available on early-stage breast tumors, it is not surprising that the search for breast cancer-specific methylation biomarkers has barely been translated into new reliable screening tests for the women at risk.

To fill the gaps in this area of research, we have analyzed the global methylation profile of DCIS, a premalignant breast lesion with a high potential to progress toward invasive and metastatic carcinoma, through loss of the myoepithelial cell layer [26]. For DNA methylation analysis, we have utilized a high-throughput methodology, recently developed in our laboratory. The methylated-CpG island recovery assay (MIRA) is a very sensitive technique that exploits the strong affinity of the MBD2/MBD3L1 complex to double-stranded CpG-methylated DNA and allows one to detect cell-type-dependent differences in DNA methylation on a microarray platform [27,28]. We have applied this technology to identify a series of novel candidate tumor suppressor genes and potential DNA methylation biomarkers in DCIS. The present study provides an unprecedented snapshot of the global methylation profile in early-stage breast carcinoma (approximately 28,000 CpG islands were analyzed) and may lead to more accurate diagnostic tests for the prediction of breast cancer. Moreover, this work draws attention to the potential role of DNA methylation in the misregulation of homeobox genes during breast tumorigenesis. Homeobox-containing transcription factors control vital functional networks during tissue development and differentiation, and their aberrant expression has been often associated, in the mammary gland, with both morphological abnormalities and oncogenesis [29-31].

## Materials and methods

### Specimens

Breast cancer samples of different histological type and grade were obtained from the City of Hope frozen tumor bank (City of Hope, Duarte, CA, USA). Tumors were staged according to the American Joint Committee on Cancer staging system protocol [32]. Tissue sections, derived from various DCIS specimens, were stained with H&E and were reviewed by a pathologist to confirm the presence and the extent of the lesions. Breast tissue obtained from non-neoplastic breast quadrants served as the normal control. All patients gave written informed consent and authorization for use of biological specimens. The present study was approved by the Institutional Review Board of the City of Hope Medical Center (IRB# 97134).

### Methylated-CpG island recovery assay-assisted microarray analysis

The MIRA and microarray analysis were performed as previously described with some modifications [27,28]. Genomic DNA was isolated from intraductal carcinomas (DCIS) and from matching normal tissues using either standard phenol–chloroform extraction methods or the DNeasy purification kit (Qiagen, Valencia, CA, USA). Between 0.5 and 1 μg DNA were double-digested with *Mse*I (5'-TTAA) and *Hha*I (5'-GCGC), to allow selective collection of substantially methylated CpG islands. Digested DNA was then incubated with a mixture of purified GST-tagged MBD2b and His-tagged MBD3L1 proteins (1:1) preincubated and bound to MagneGST glutathione particles (TM240; Promega, Madison, WI, USA). Binding reactions were carried out overnight at 4°C on a rocking platform, in 1× MIRA binding buffer (10 mM Tris–HCl (pH 7.5), 50 mM NaCl, 1 mM ethylenediamine tetraacetic acid, 1 mM dithiothreitol, 3 mM MgCl_2_, 0.1% Triton-X100, 5% glycerol, 25 μg/ml BSA, and sonicated JM110 (*dcm *minus) bacterial DNA). Pelleted glutathione beads were washed three times with a high salt-washing buffer (containing 700 mM NaCl), and the methylated DNA-enriched genomic DNA fraction was eluted with a guanidinium hydrochloride-containing buffer. DNA purification was carried out using the Qiaquick PCR purification kit according to the manufacturer's instructions (Qiagen).

Following the MIRA pulldown, CpG-enriched DNA fragments were ligated to *Mse*I linkers (upper strand sequence, 5'-AGCAACTGTGCTATCCGAGGGAT; lower strand sequence, 5'-TAATCCCTCGGA) and were PCR-amplified for up to 20 cycles by real-time PCR. Two micrograms each of the amplicons from MIRA-enriched tumor DNA and from MIRA-enriched normal control samples were labeled with Cy5-dCTP and Cy3-dCTP respectively (GE Healthcare Bio-Sciences Corp., Piscataway, NJ, USA), using a BioPrime Array CGH Genomic Labeling kit (Invitrogen, Carlsbad, CA, USA). The purified samples were then mixed, and hybridized to CpG island microarrays, according to the Agilent ChIP-on-chip protocol (version 9.0; Agilent Technologies, Santa Clara, CA, USA). Human CpG island microarrays contain 237,000 oligonucleotide probes covering 27,800 CpG islands and were purchased from Agilent Technologies (Santa Clara, CA, USA).

### Microarray data analysis

Following hybridization and washing (Agilent ChIP-on-chip protocol, version 9.0), microarray slides were scanned using an Axon GenePix 4000b scanner (Molecular Devices, Sunnyvale, CA, USA) and images were quantified by GenePix Pro 6 software (Molecular Devices, Sunnyvale, CA, USA). Preprocessing of raw data and statistical analysis were performed as previously described with some modifications [33]. Log_2_-transformed ratios on each array were analyzed separately.

Probes were selected as positive if their ratios fell into more than the 98th percentile range on the array. Methylation regions (peaks) were defined as regions that contain a minimum of three positive probes. One negative probe was allowed within the peak as long as it was not present at the end of the region. The average of the log_2 _ratios of the positive probes within each peak was assigned as the peak score. Peaks were then annotated based on their location relative to known transcripts (UCSC hg18 refseq). Peaks overlapping with the ±1,000 bp region of a known transcription start site to -10 kb of the transcription start site were annotated as upstream of the transcript, and peaks that overlap with the ±1,000 bp region of a known transcription termination site to +10 kb downstream of the transcription termination site were annotated as downstream of the transcript. Peaks falling into known transcripts but not within 1,000 bp of either the transcription start site or the transcription termination site were denoted intragenic.

The transcripts that have either upstream, intragenic or downstream peaks in at least three out of six DCIS samples were selected as interesting targets, and are reported in Table 1. For peaks that fell into uncharacterized regions of the genome, the location of the CpG island was used instead (Table 2). Microarray data were deposited in the Gene Expression Omnibus repository [GEO:GSE14865].

**Table 1:** Methylated target genes identified by methylated-CpG island recovery assay-assisted microarray analysis

Target number	Gene	Description	Gene location	Strand	Position relative to gene^a^	Average ratio^b^	Count
1*	FOXE1	Forkhead box E1	Chr9: 99655357 to 99658818	+	Downstream	13.76	4
2	SEMA6C	Semaphorin 6C	Chr1: 149370786 to 149385728	-	Downstream	6.40	4
3*	**HNF1B**^c^	Hnf1 homeobox B	Chr17: 33120546 to 33179209	-	Upstream	6.06	4
4	**OTX1**	Orthodenticle homeobox 1	Chr2: 63131468 to 63137816	+	Intragenic	4.83	4
5*	**TLX1**	T-cell leukemia homeobox 1	Chr10: 102881050 to 102887535	+	Intragenic	4.50	4
6	ESAM	Endothelial cell adhesion molecule	Chr11: 124128228 to 124137433	-	Intragenic	4.16	4
7*	CNTNAP1	Contactin associated protein 1	Chr17: 38088158 to 38105358	+	Intragenic	3.94	4
8*	GFI1	Growth factor independent 1 transcription repressor	Chr1: 92712905 to 92725021	-	Intragenic	3.64	4
9*	RASL10A	Ras-like, family 10, member A	Chr22: 28038921 to 28041748	-	Intragenic	3.42	4
10*	IFNA8	Interferon, alpha 8	Chr9: 21399146 to 21400184	+	Upstream	3.41	4
11	ERGIC2	Ergic and golgi 2	Chr12: 29384845 to 29425410	-	Upstream	3.39	4
12	PCDH7	Protocadherin 7	Chr4: 30331134 to 30757519	+	Intragenic	3.16	4
13	RASGRP2	Ras guanyl releasing protein 2	Chr11: 64250958 to 64269504	-	Intragenic	3.01	4
14	NFATC1	Nuclear factor of activated T-cells 1	Chr18: 75256759 to 75390311	+	Intragenic	2.93	4
15	RLTPR	Rgd motif, leucine-rich repeats, tropomodulin domain and proline-rich containing gene	Chr16: 66236530 to 66248973	+	Intragenic	2.75	4
16	AK123344	Hypothetical gene	Chr10: 102979341 to 102985256	+	Downstream	16.49	3
17	C1orf114	Chromosome 1 open reading frame 114	Chr1: 167630737 to 167663294	-	Upstream	11.13	3
18	TRPS1	Trichorhinophalangeal syndrome I	Chr8: 116489899 to 116750402	-	Intragenic	11.07	3
19	PCDHGA12	Protocadherin gamma subfamily A, 12	Chr5: 140790341 to 140872730	+	Upstream	11.02	3
20	C14orf25	Chromosome 14 open reading frame 25	Chr14: 37150207 to 37580397	+	Upstream	10.34	3
21	CDKN2A	Cyclin-dependent kinase inhibitor 2A (P16)	Chr9: 21957750 to 21984490	-	Downstream	9.86	3
22	SCRT2	Scratch homolog 2, zinc finger protein	Chr20: 590240 to 604823	-	Downstream	9.47	3
23	BCOR	Bcl6 co-repressor	Chrx: 39795443 to 39921526	-	Upstream	9.43	3
24	KHDC1	Kh homology domain containing 1	Chr6: 74007759 to 74076659	-	Upstream	9.00	3
25*	NXPH1	Neurexophilin 1	Chr7: 8440109 to 8759118	+	Intragenic	8.83	3
26	CNR1	Cannabinoid receptor 1 (brain)	Chr6: 88910156 to 88932281	-	Upstream	8.78	3
27	BC039088	Hypothetical gene	Chr5: 43050280 to 43054670	-	Upstream	8.61	3
28	**EVX2**	Even-skipped homeobox 2	Chr2: 176653080 to 176656936	-	Downstream	8.43	3
29*	MT1E	Metallothionein 1E	Chr16: 55217085 to 55218525	+	Upstream	8.36	3
30*	NR2F2	Nuclear receptor subfamily 2, group F, member 2	Chr15: 94674950 to 94683047	+	Downstream	8.24	3
31	**HOXC13**	Homeobox C13	Chr12: 52618843 to 52626595	+	Upstream	8.08	3
32	**HOXD8**	Homeobox D8	Chr2: 176702722 to 176704974	+	Upstream	7.97	3
33	SYCP2L	Synaptonemal complex protein 2-like	Chr6: 10995049 to 11082527	+	Upstream	7.58	3
34	PCDHGB6	Protocadherin gamma subfamily B, 6	Chr5: 140767953 to 140872730	+	Intragenic	7.37	3
35	ACTA1	Actin, alpha 1, skeletal muscle	Chr1: 227633615 to 227636466	-	Upstream	7.36	3
36*	PRDM14	Pr domain containing 14	Chr8: 71126576 to 71146116	-	Intragenic	7.27	3
37*	**HOXB13**	Homeobox B13	Chr17: 44157124 to 44161110	-	Intragenic	6.91	3
38	**OTX2**	Orthodenticle homeobox 2	Chr14: 56337177 to 56346937	-	Upstream	6.70	3
39	ZNF711	Zinc finger protein 711	Chrx: 84385652 to 84415025	+	Upstream	6.63	3
40*	NR2E1	Nuclear receptor subfamily 2, group E, member 1	Chr6: 108593954 to 108616706	+	Intragenic	6.44	3
41*	TAC1	Tachykinin, precursor 1	Chr7: 97199206 to 97207720	+	Upstream	6.37	3
42*	CPEB1	Cytoplasmic polyadenylation element binding protein 1	Chr15: 81009005 to 81113783	-	Upstream	5.99	3
43	NKAPL	Nfkb activating protein-like	Chr6: 28335076 to 28336715	+	Upstream	5.86	3
44	**NKX6-2**	Nk6 homeobox 2	Chr10: 134448309 to 134449527	-	Downstream	5.72	3
45*	TGIF2	Tgfb-induced factor homeobox 2	Chr20: 34635423 to 34655766	+	Upstream	5.60	3
46	CR596471	Hypothetical gene	Chrx: 133511720 to 133522094	+	Upstream	5.56	3
47	**EVX2**	Even-skipped homeobox 2	Chr2: 176653080 to 176656936	-	Intragenic	5.55	3
48	AX747981	Hypothetical gene	Chr8: 96148213 to 96154324	-	Upstream	5.48	3
49	ST8SIA3	St8 alpha-*N*-acetyl-neuraminide alpha-2,8-sialyltransferase 3	Chr18: 53170718 to 53187159	+	Intragenic	5.47	3
50	WT1	Wilms tumor 1	Chr11: 32365900 to 32413663	-	Intragenic	5.40	3
51	**BARHL2**	Barh-like homeobox 2	Chr1: 90950167 to 90955382	-	Upstream	5.39	3
52	**IRX1**	Iroquois homeobox 1	Chr5: 3649167 to 3654517	+	Intragenic	5.39	3
53	KLF11	Kruppel-like factor 11	Chr2: 10101132 to 10112414	+	Upstream	5.26	3
54	**HLXB9**	Homeobox Hb9	Chr7: 156479507 to 156496108	-	Upstream	5.25	3
55	**NKX2-8**	Nk2 homeobox 8	Chr14: 36118966 to 36121537	-	Upstream	5.20	3
56	ZFP91	Zinc finger protein 91 homolog (mouse)	Chr11: 58103162 to 58145091	+	Upstream	5.07	3
57	**LHX2**	Lim homeobox 2	Chr9: 125813709 to 125835263	+	Intragenic	5.04	3
58	**MEIS1**	Meis homeobox 1	Chr2: 66516036 to 66653085	+	Downstream	5.00	3
59*	**PAX2**	Paired box 2	Chr10: 102495457 to 102579688	+	Intragenic	4.95	3
60*	**LHX9**	Lim homeobox 9	Chr1: 196153139 to 196165896	+	Intragenic	4.81	3
61	GRASP	Grp1-associated scaffold protein	Chr12: 50687014 to 50695938	+	Upstream	4.78	3
62	**LHX8**	Lim homeobox 8	Chr1: 75366706 to 75399806	+	Intragenic	4.77	3
63	**HOXD12**	Homeobox D12	Chr2: 176672775 to 176673734	+	Upstream	4.66	3
64	**PAX5**	Paired box 5	Chr9: 36828530 to 37024476	-	Intragenic	4.56	3
65	**LBX1**	Ladybird homeobox 1	Chr10: 102976722 to 102978707	-	Downstream	4.53	3
66	EGFR	Epidermal growth factor receptor	Chr7: 55054218 to 55242525	+	Upstream	4.45	3
67	ODZ3	Odz, odd Oz/ten-m homolog 3	Chr4: 183302134 to 183508463	+	Upstream	4.40	3
68	TM7SF4	Transmembrane 7 superfamily member 4	Chr8: 105421230 to 105438092	+	Upstream	4.22	3
69	ZNF311	Zinc finger protein 311	Chr6: 29070573 to 29081014	-	Downstream	4.09	3
70	PCDH19	Protocadherin 19	Chrx: 99433297 to 99551927	-	Intragenic	4.06	3
71	FLJ45983	Hypothetical gene	Chr10: 8132418 to 8135453	-	Upstream	3.84	3
72	AX747375	Hypothetical gene	Chr19: 41955897 to 41958529	+	Downstream	3.83	3
73	PPP2R2C	Protein phosphatase 2 regulatory subunit B, gamma isoform	Chr4: 6373205 to 6525227	-	Intragenic	3.58	3
74	**EMX1**	Empty spiracles homeobox 1	Chr2: 72998111 to 73015528	+	Intragenic	3.57	3
75	PRKCSH	Protein kinase C substrate 80K-H	Chr19: 11407268 to 11422782	+	Upstream	3.56	3
76	FZD1	Frizzled homolog 1	Chr7: 90731718 to 90736068	+	Intragenic	3.52	3
77	**LBX2**	Ladybird homeobox 2	Chr2: 74578151 to 74583951	-	Upstream	3.42	3
78	**HOXC13**	Homeobox C13	Chr12: 52618842 to 52626595	+	Intragenic	3.40	3
79	MT1A	Metallothionein 1A	Chr16: 55230078 to 55231500	+	Upstream	3.36	3
80	**DLX5**	Distal-less homeobox 5	Chr7: 96487637 to 96492079	-	Intragenic	3.06	3
81	LDOC1L	Leucine zipper, downregulated in cancer 1-like	Chr22: 43267113 to 43272669	-	Intragenic	2.30	3

^a^For definitions of upstream, intragenic, and downstream, see Materials and methods (Microarray data analysis). The transcripts that have either upstream, intragenic or downstream peaks in at least three out of six DCIS samples were selected as positive targets. *Gene targets verified by combined bisulfite restriction analysis assays or bisulfite sequencing. ^b^Average of the log_2 _ratios assigned to positive peaks across the six DCIS. ^c^Homeobox genes are indicated in bold.

**Table 2:** Methylated CpG islands not associated with known genes

Target number	Symbol	CpG island location^a^	Average ratio^b^	Count
1	CGI 1:42	Chr1: 38714506 to 38714991	6.27	4
2	CGI 6:60	Chr6: 106535804 to 106536465	4.69	4
3	CGI 2:112	Chr2: 239419845 to 239420943	4.22	4
4	CGI 4:34	Chr4: 174658599 to 174659018	2.94	4
5*	CGI 7:48	Chr7: 35460738 to 35461227	17.26	3
6	CGI 8:121	Chr8: 100054909 to 100056159	16.81	3
7	CGI 10:46b	Chr10: 119484483 to 119484981	15.44	3
8	CGI 17:16	Chr17: 44182434 to 44182640	13.96	3
9	CGI 7:351	Chr7: 129205522 to 129209745	11.56	3
10	CGI 6:19	Chr6: 27755772 to 27755984	10.16	3
11	CGI 8:31	Chr8: 81968510 to 81968882	8.45	3
12	CGI 22:48	Chr22: 44655029 to 44655728	7.98	3
13	CGI 2:77	Chr2: 176639821 to 176640909	7.91	3
14	CGI 5:47	Chr5: 176039674 to 176040127	7.78	3
15	CGI 7:29	Chr7: 129210233 to 129210591	7.60	3
16	CGI 3:47	Chr3: 134875805 to 134876344	6.58	3
17*	CGI 4:35	Chr4: 24699204 to 24699608	6.22	3
18	CGI 6:18	Chr6: 27571155 to 27571358	6.18	3
19	CGI 7:277	Chr7: 154857318 to 154860615	5.91	3
20	CGI 10:46	Chr10: 102409137 to 102409658	5.84	3
21	CGI 4:21	Chr4: 174657922 to 174658134	5.53	3
22	CGI X:92	Chrx: 136459742 to 136460985	4.81	3
23	CGI 22:18	Chr22: 47762399 to 47762664	4.15	3
24	CGI 1:130	Chr1: 199774402 to 199775940	3.77	3
25	CGI 5:26	Chr5: 3379328 to 3379645	3.42	3
26	CGI 5:87	Chr5: 134853297 to 134854395	3.16	3
27	CGI 8:72	Chr8: 1100465 to 1101480	2.35	3

^a^For uncharacterized gene targets, CpG island location was assigned instead of gene location. ^b^Average of the log_2 _ratios assigned to positive peaks across the six DCIS. Targets with methylated peaks within the CpG island in at least three out of six DCIS samples were considered significant. *Unknown CpG islands verified by combined bisulfite restriction analysis assays or bisulfite sequencing.

### Combined bisulfite restriction analysis and bisulfite sequencing

Total genomic DNA was isolated from human breast carcinoma and from normal tissues as described above. To analyze the methylation status of cytosines in several hypermethylated microarray targets, DNA (1 μg) was treated with sodium bisulfite according to the manufacturer's protocol (EpiTect; Qiagen) and was subjected to combined bisulfite restriction analysis [34]. The PCR primer sequences used to amplify the candidate genes in bisulfite-treated DNA are available upon request. HeLa DNA was methylated *in vitro *with M. SssI methyltransferase and served as a positive control. For sequence analysis, the PCR products obtained after bisulfite conversion were cloned into the TOPO-TA cloning vector (Invitrogen) and up to 12 individual clones were sequenced.

## Results

### Genome-wide detection of methylated CpG islands in DCIS

The MIRA, used in combination with microarray analysis, is a high-resolution mapping technique and has proven successful in profiling global DNA methylation patterns in lung cancer [33,35]. In the present study we have applied this sensitive method to establish the methylation status of CpG islands in early-stage ductal carcinoma and to investigate its potential role in the initiation and development of breast cancer. Six DCIS were screened for methylation by MIRA-based microarrays. To ensure consistency with the initial clinical diagnosis, and the presence of epithelial cells in the sample, histological examination of the specimens (both cancer samples and their matching normal) was conducted with the help of a pathologist.

**Figure 1:**
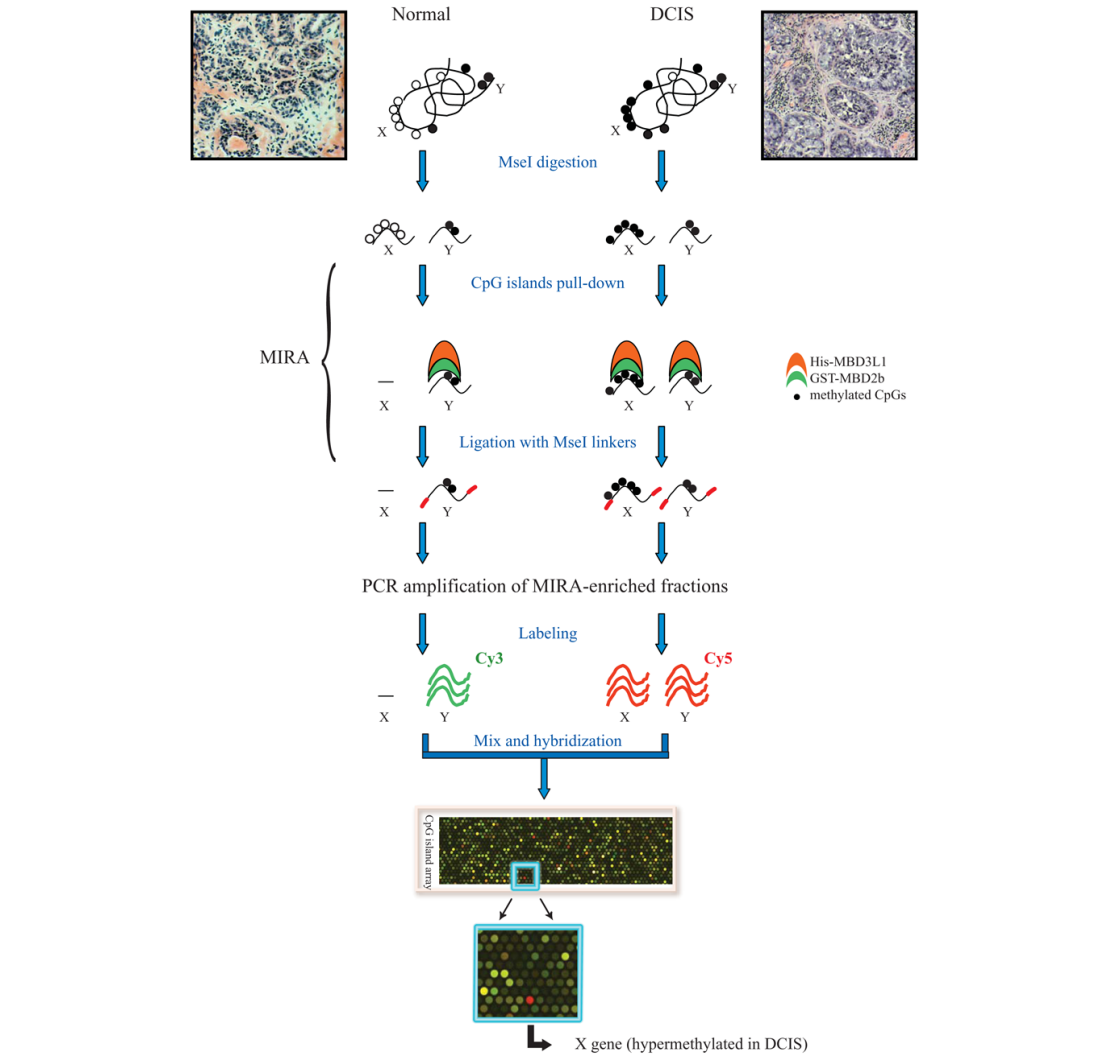
Outline of the methylated-CpG island recovery assay-assisted CpG island microarray analysis. Prior to microarray analysis, tissue sections, derived from six independent ductal carcinomas *in situ *(DCIS) and their matching normal areas, were stained with H&E and validated by a pathologist. Genomic DNA derived from tumor specimens and matching normal tissue was then subjected to methylated-CpG island recovery assay (MIRA) pull-down as described in Materials and methods. MIRA-enriched fractions were labeled with different dyes, mixed, and hybridized to CpG island-Agilent slides and the relative enrichment factors between different tissues were determined by statistical analysis.

The H&E slides derived from tumor samples displayed a predominant DCIS component (70% to 80%) over normal stromal tissue and infiltrating lymphocytes (as shown for DCIS number 4 in Figure 1, upper right panel). Since contamination with low amounts of normal cells is not expected to interfere with the overall interpretation of the methylation data, tumor specimens were not subjected to further microdissection. Normal healthy breast tissues adjacent to the lesions and obtained at the time of the surgical resection were used as a control. When matching normal tissue was not available, a DNA mixture from several normal breast tissues was used as a control. To avoid epigenetic variations due to age, reproductive state and/or cancer stage, we pooled together, whenever possible, DNA of normal breast tissues derived from comparable donors (same age frame, tumor stage, estrogen receptor/progesterone receptor status, and so forth).

Genomic DNA isolated from DCIS and normal tissue was subjected to MIRA enrichment, labeling, and subsequent microarray analysis, as depicted in detail in Figure 1. DCIS-specific methylation regions (peaks) were defined using stringent statistical criteria, and a full description of the algorithm used for the analysis is provided in Materials and methods. Using this approach, we generated a list of over 100 significant CpG islands that display aberrant methylation in early-stage breast cancer and were methylation-positive in at least three out of six DCIS examined (Tables 1 and 2). Consistent with a potential role in transcriptional silencing, anomalous levels of DNA methylation were detected at the 5' end of known genes, in proximity of promoter regions or further upstream. Numerous CpG islands mapping to intragenic or downstream regions of annotated genes, however, were also found to be heavily methylated in DCIS (Table 1).

### Verification of tumor-specific DNA methylation by combined bisulfite restriction analysis

We next confirmed tumor-specific methylation for several of the targets identified through array analysis using the *Bst*UI combined bisulfite restriction analysis (COBRA) assay. In this assay, bisulfite-converted DNA is PCR-amplified using gene-specific primers and is then digested with the restriction endonuclease *Bst*UI, which recognizes the sequence 5'-CGCG. Unmethylated restriction sites are converted to 5'-TGTG by sodium bisulfite and PCR and resist *Bst*UI digestion, whereas methylated sites remain unchanged and are cleaved by the enzyme. The digested fragments displayed on agarose gels are thus indicative of methylated *Bst*UI sites in the region analyzed.

**Figure 2:**
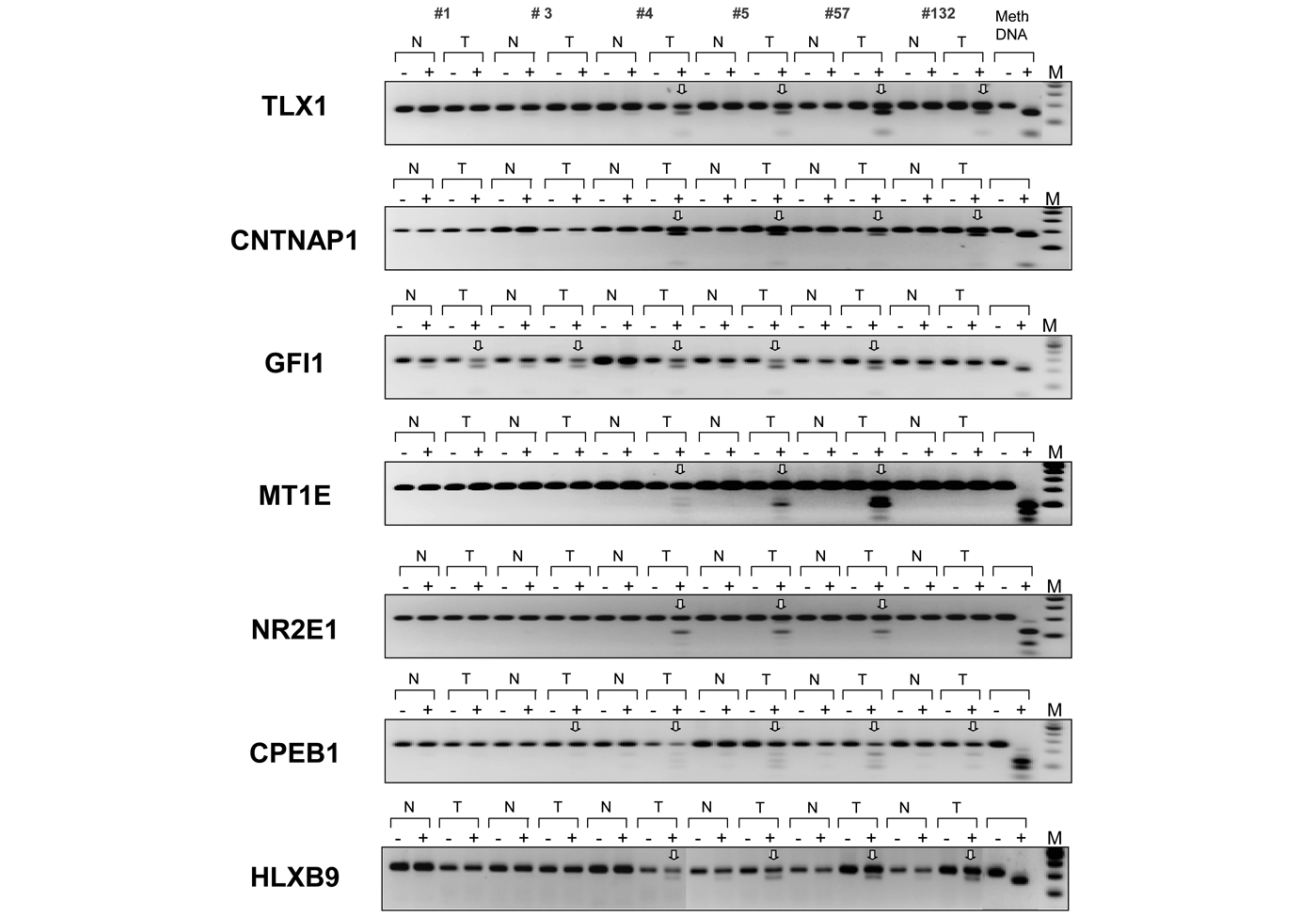
Verification of tumor-specific methylation of seven candidate target genes. These targets were identified by the MIRA-assisted microarray approach (target numbers 5, 7, 8, 29, 40, 42 and 54, corresponding to *TLX1*, *CNTNAP1*, *GFI1*, *MT1E*, *NR2E1*, *CPEB1 *and *HLXB9*, respectively; Table 1). Genomic DNA from ductal carcinomas *in situ *(T) and matching normal breast tissues (N) was treated with sodium bisulfite and the target CpG island sequences were amplified using gene-specific primers. Methylation was confirmed by a *Bst*UI combined bisulfite restriction analysis assay, which produces digestion products when *Bst*UI restriction sites are methylated and not converted by bisulfite. HeLa DNA was methylated *in vitro *with the SssI methyltransferase and served as a positive control (Meth. DNA, Methylated HeLa DNA). Vertical white arrows indicate hypermethylated alleles in the target sequence. ±, digestion was carried out with or without *Bst*UI restriction enzyme; N/T, normal/tumor pairs. When matching normal tissue was not available, a DNA mixture derived from several normal breast tissues was used instead. M, DNA marker.

Representative examples of COBRA results are shown in Figure 2. Here, we have inspected the methylation status of the candidate genes ranked numbers 5, 7, 8, 29, 40, 42 and 54 on the list of differentially methylated targets (corresponding to T-cell leukemia homeobox 1 (*TLX1*), *CNTNAP1*, *GFI1*, *MT1E*, *NR2E1*, *CPEB1 *and homeobox HB9 (*HLXB9*), respectively). All seven CpG islands appear methylated with high specificity (no or very little methylation detected in normal breast tissue) in the DCIS analyzed, and with methylation frequencies ranging from 50% to 83% depending on the target gene. No target region scrutinized so far exhibited robust CpG methylation across all six intraductal carcinomas (Table 1 and data not shown). Interestingly, we noticed that one-third of the CpG islands identified by microarray analysis (26 out of the 81 annotated hits) are associated with members of various homeobox superfamilies (*HOX, LHX, NKX, PAX*, and so forth) and are preferential targets of *de novo *methylation in early-stage breast cancer. These master regulators control vital functional networks during tissue development and differentiation, and are misregulated in a variety of malignancies, including breast cancer [29,36].

**Figure 3:**
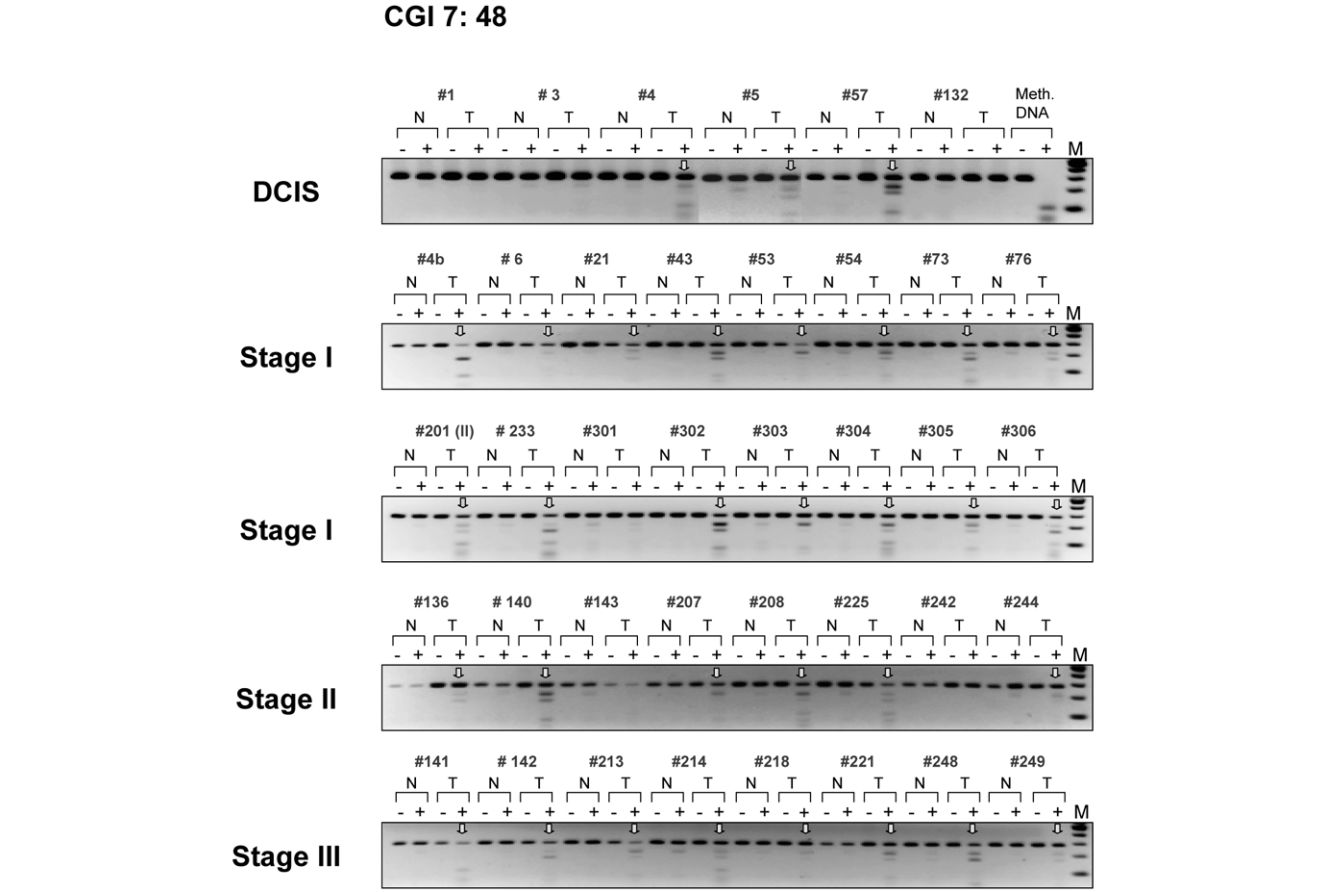
Methylation of an uncharacterized CpG island on chromosome 7. Six cases of ductal carcinoma *in situ *(DCIS) and 32 invasive breast tumors of different histological type and grade were analyzed for CpG methylation. The target CpG island (hit number 5, Table 2) was subjected to the *Bst*UI combined bisulfite restriction analysis assay. N/T, normal/tumor pairs. Vertical arrows indicate tumor-specific methylation. Target number 201 is a stage II breast carcinoma. M, DNA marker; Meth. DNA, HeLa DNA methylated *in vitro *with SssI methyltransferase, serving as a positive control.

**Figure 4:**
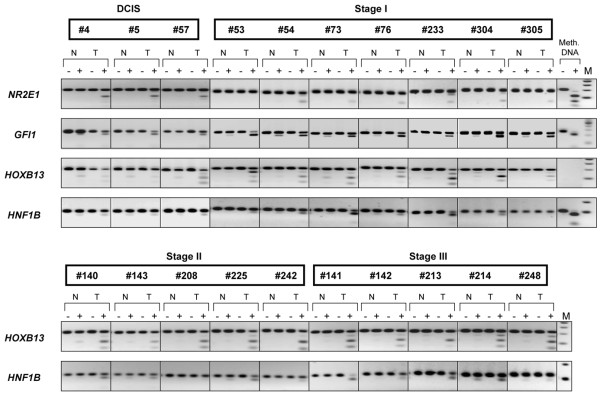
Combined bisulfite restriction analysis in ductal carcinoma *in situ *and more advanced primary breast tumors. The *NRN1*, *GFI1*, *HOXB13*, *HNF1B*-associated CpG islands were analyzed by Combined bisulfite restriction analysis in ductal carcinoma *in situ *(DCIS) and more advanced breast carcinoma. Three representative DCIS, seven stage I tumors, five stage II tumors and five stage III breast tumors are shown. The total number of specimens analyzed per gene and the relative methylation frequencies are presented in Table 3. N/T, normal/tumor pairs; M, DNA marker; Meth. DNA, HeLa DNA methylated *in vitro *with SssI methyltransferase, serving as a positive control.

To explore this interesting finding in more detail, we then focused our attention on several homeobox genes. Besides the CpG islands associated with the *TLX1 *gene and with the *HLXB9 *gene (Figure 2), we have examined the methylation status of the *HNF1B *and *HOXB13 *candidate gene targets (hit numbers 3 and 37, respectively). An uncharacterized CpG island located on chromosome 7 (CGI 7:48 and hit number 5 in Table 2) was also selected for COBRA analysis. Although some levels of methylation could be detected in normal tissues, methylation of *HOXB13*, *HNF1B*, and CGI 7:48 CpG islands was clearly more pronounced in intraductal carcinomas (Figure 3, upper panel and Figure 4). Considering that the same matching controls were negative in other COBRA assays (Figure 2), we can exclude that the methylation signal observed here in normal samples is due to contamination with neighboring cancer cells. Rather, distinct cell populations within the normal breast tissue may display specific DNA methylation profiles, as recently established [37]. Differential promoter hypermethylation in benign breast epithelium derived from cancer patients can also be a function of age in a gene-specific manner [16,38]. Likewise, the occurrence of unmethylated alleles in the cancer samples may reflect the heterogeneity of CpG methylation within the cell populations of the tumor itself, but also the presence of normal cells in the specimens.

### Confirmation of the methylation pattern in invasive breast tumors

**Figure 5:**
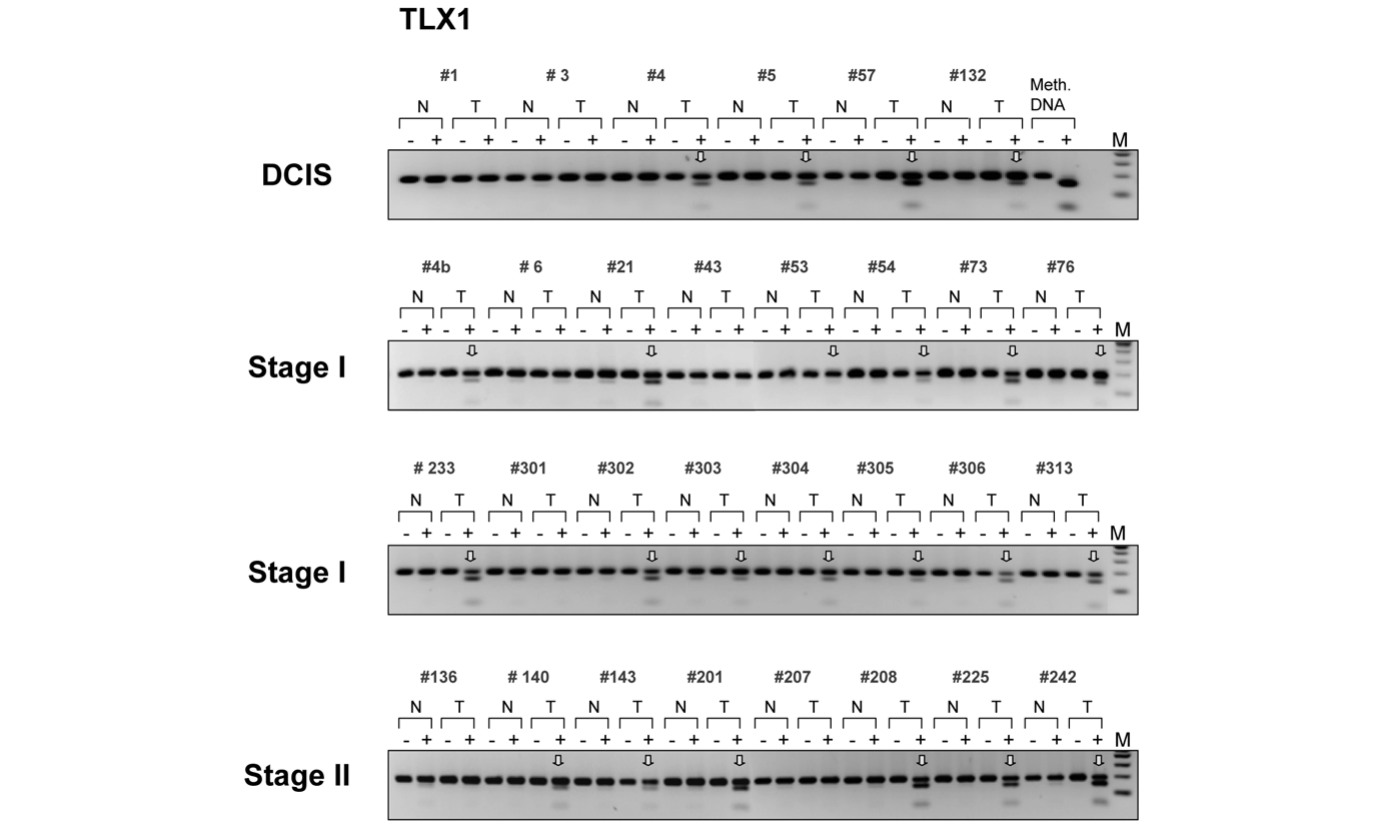
Methylation of the *TLX1 *gene in ductal carcinoma *in situ *and invasive primary breast tumors. Six cases of ductal carcinoma *in situ *(DCIS), 16 cases of stage I tumors, and eight cases of stage II tumors were analyzed. After sodium bisulfite treatment, the CpG island within the *TLX1 *gene was amplified with suitable primers and subjected to the *Bst*UI combined bisulfite restriction analysis assay. Digested fragments on the gel are indicative of methylated *Bst*UI restriction sites (5'-CGCG) within the CpG island. Vertical arrows indicate tumor-specific methylation in the target sequence. N/T, normal/tumor pairs; M, DNA marker; Meth. DNA, HeLa DNA methylated *in vitro *with SssI methyltransferase, serving as a positive control.

To corroborate the DCIS-specific methylation profiles of *TLX1*, CGI 7:48, *HOXB13*, and *HNF1B*, we then extended the COBRA analysis to a series of primary breast tumors of different histological type and stage (Figures 3 and 5, lower panels and Figure 4). The *TLX1 *CpG island was methylated in 13 out of the 16 stage I breast tumors (81%) and in six out of the eight stage II invasive carcinomas (75%). Likewise, the CpG island located on chromosome 7 (CGI 7:48) was methylated in almost every stage I tumor examined (93%) and in 15 out of the 17 more advanced tumors (stages II and III tumors, 88%). Of the 32 invasive breast carcinomas tested (15 cases of stage I tumors, nine cases of stage II tumors and eight cases of stage III tumors) 29 displayed higher levels of methylation relative to their matching controls within the *HOXB13 *element (91%), whereas the *HNF1B *CpG island was methylated in 21 tumor samples (66%) (partial COBRA data are shown in Figure 4). Stage I tumors already exhibited a significant degree of methylation (87% for *HOXB13 *and 73% for *HNF1B*), confirming the rapid and early nature of epigenetic reprogramming in breast cancer (Figure 4, representative COBRA analysis and Table 3).

**Table 3:** Methylation frequencies of selected genes in ductal carcinoma *in situ *and invasive breast tumors

	Ductal carcinoma *in situ*	Stage I tumors	Stage II tumors	Stage III tumors
*TLX1*	4/6 (67%)	13/16 (81%)	6/8 (75%)	
*CGI 7:48*	3/6 (50%)	14/15 (93%)	7/9 (78%)	8/8 (100%)
*HOXB13*	3/6 (50%)	13/15 (87%)	8/9 (89%)	8/8 (100%)
*HNF1B*	3/6 (50%)	11/15 (73%)	5/9 (56%)	5/8 (63%)
*GFI1*	5/6 (83%)	9/12 (75%)	7/8 (88%)	
*NR2E1*	3/6 (50%)	8/15 (53%)		

Rodriguez and colleagues have reported recently that hypermethylation of the *HOXB13 *gene is a late event in breast tumorigenesis [39]. This apparent discrepancy with our results can be ascribed to the different methods used for the analysis (methylation-specific PCR versus our genome-wide DNA methylation profiling), the different specimens examined in the two studies (invasive-stage breast carcinomas versus DCIS), and the different location of the CpG islands on the *HOXB13 *gene (promoter versus an intragenic CpG island, target number 37). Methylation of this intragenic CpG island is an early event in breast cancer development and may precede promoter methylation. Whether this intragenic CpG island affects the *HOXB13 *gene expression remains to be seen. Two additional targets, the growth factor transcriptional repressor *GFI1 *gene (target number 8) and the nuclear receptor *NR2E1 *gene (target number 40), were inspected to evaluate the level of methylation in early-stage breast carcinomas. In agreement with the MIRA results, we found that the *GFI1 *CpG island was hypermethylated in 14 out of the 18 tumors examined (six DCIS and 12 stage I tumors, 78%) while the *NR2E1 *target region was methylated in 11 out of the 21 early-stage breast tumors (six DCIS and 15 stage I tumors, 52%) (Figure 4, partial data and Table 3).

**Figure 6:**
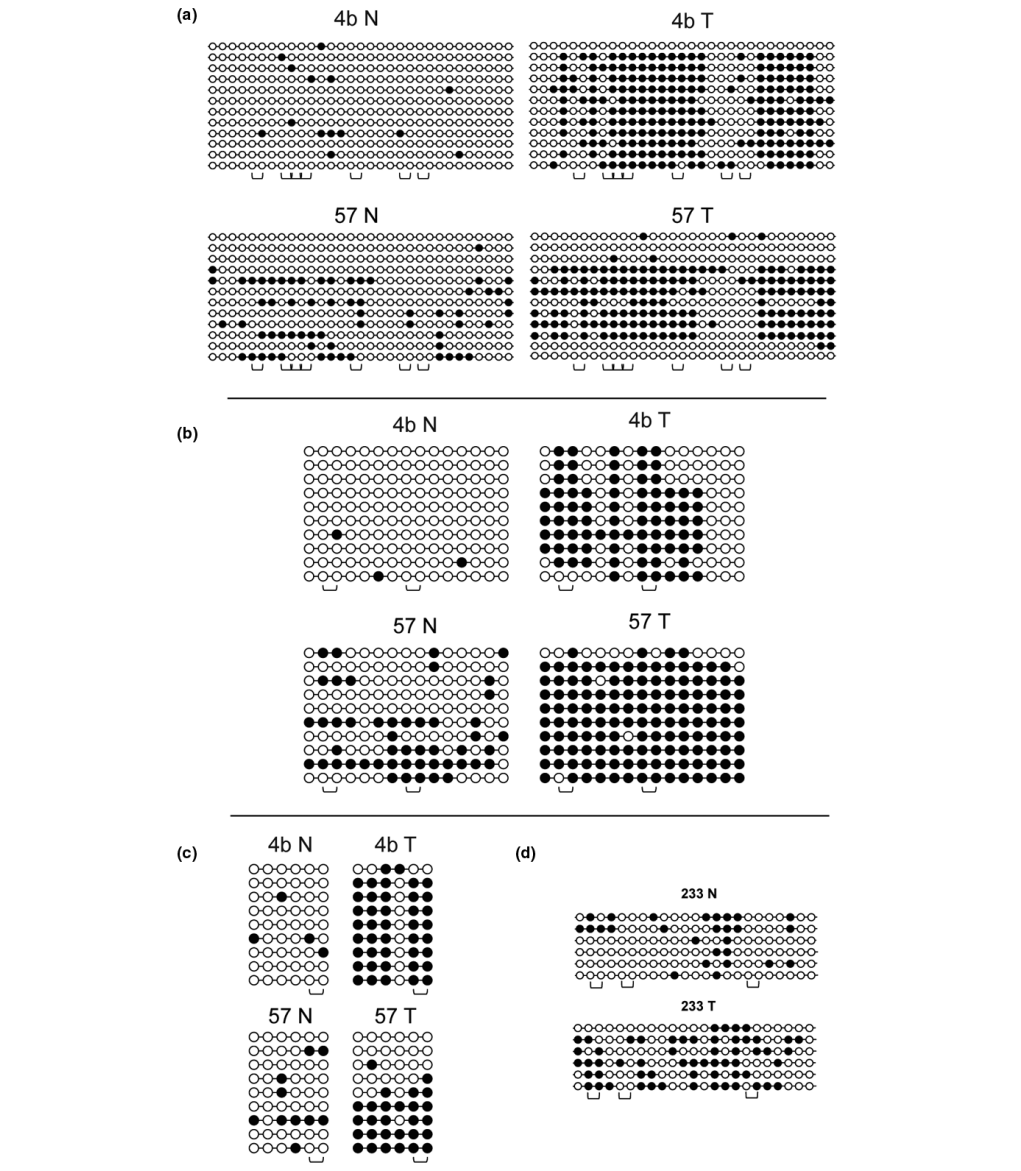
Bisulfite sequencing data. The extent of CpG methylation was determined for target sequences within **(a) **CGI 7:48, **(b) ***HOXB13*, **(c) ***HNF1B*, and **(d) ***HLXB9 *by sodium bisulfite sequencing. Primary breast tumors were subjected to bisulfite DNA sequencing together with their matching normal counterpart (ductal carcinoma *in situ *case number 57 and stage I, case numbers 4b and 233). Sequencing results of several independent clones are shown. Black circles, methylated CpG dinucleotides. Open brackets indicate the location of the *Bst*UI restriction sites in the target sequence.

To assess the extent of CpG methylation within the *HOXB13*, *HNF1B*, *HLXB9 *and CGI 7:48 target sequences, primary breast tumors were also subjected to bisulfite DNA sequencing together with their matching normal tissues (DCIS case number 57 and stage I case numbers 4b and 233). As expected, there is an evident tendency towards increased methylation in tumor-derived samples; the occurrence of early-stage cancer-specific methylated CpGs is very significant (*P *< 0.001, Fisher's exact test) (Figure 6).

## Discussion

DCIS is suspected to be a direct, although not obligate, precursor of invasive breast cancer, and aberrant DNA methylation is believed to play a crucial role in breast tumorigenesis. Considering that epigenetic changes often become apparent in early phases of the disease, we speculated that the identification of DCIS-specific methylated biomarkers might be crucial to elucidate the molecular mechanisms underlying the initiation and development of breast cancer and to conceive effective strategies for early diagnosis.

To acquire valuable information into the epigenetic switches that may promote and/or contribute to the initial neoplastic events, we have analyzed the DNA methylation profile of DCIS, on a MIRA-based CpG island microarray platform. This novel and sensitive genome-wide screening approach has led to the identification of 108 CpG islands that display aberrant levels of DNA methylation in early breast lesions. Of the 81 CpG islands associated with known genes, only 37 map to promoter regions or further upstream (46%). In agreement with recently published data [40], more than one-half of the methylated CpG islands in normal genomes fall within the body of the gene or in downstream regions. The functional role of these intergenic and intragenic CpG-rich elements remains obscure, but it has been suggested that they may constitute short independent transcriptional units [40].

Several targets have been examined by conventional bisulfite methods and found to be differentially methylated in multiple breast tumors, in complete agreement with the MIRA results. Most importantly, these gene candidates display methylation frequencies ranging from 50% to 83% in DCIS and up to 93% in stage I breast cancer, depending on the target gene, and these candidates hold great promise, alone or in combination, for future diagnostic applications. We were also able to identify several genes, such as *CDKN2A*, *PCDHGB6*, and *WT1 *(Table 1) well known to be methylated and transcriptionally silent in breast cancer [10,23]. Many other 'conventional' methylation markers, however, were not represented in our data set. This apparent discrepancy with previous reports can be ascribed, in part, to the different conditions utilized in microarray data analysis to define thresholds; consequently, some genes may be classified as false negative simply because they fall below the statistical cutoff points. Digestion of genomic DNA, prior to the MIRA pull down, with the methylation-sensitive endonuclease *Hha*I can also be crucial in excluding those CpG islands that are not methylated at *Hha*I restriction sites (5'-GCGC), although they are methylated at surrounding CpGs within the target sequence. In addition, genes previously described as methylated in advanced-stage breast cancer may not be methylated in DCIS.

Surprisingly, most of the targets identified in the present study have never been linked to epigenetic errors during breast carcinogenesis and may shed new light into the molecular mechanisms underlying the insurgence of breast cancer. The employment of undissected breast tissue that fails to discern the epigenetic contribution of the single cell subtypes and the restricted number of DCIS used in the microarray analysis, however, may represent important limitations to this study and need to be kept in consideration.

Apart from the potential discovery of novel tumor suppressor genes and/or methylation biomarkers, relevant for a better comprehension and management of the disease, the present study has uncovered a broad epigenetic phenomenon that occurs at the onset of breast cancer development. Interestingly, we found that 32% of the total hypermethylated CpG islands (26 out of the annotated 81 hits) are associated with members of multiple homeobox gene subfamilies – a surprisingly high percentage considering that, so far, only ~300 homeobox genes have been identified in the human genome (roughly 1% of the presumed battery of protein-coding genes) [41]. CpG methylation of homeobox genes has been sporadically observed during breast tumorigenesis; that is, methylation of the *HOXB13 *gene [39] and members of the HOXA cluster [42,43]. The extent and the recurrence of this epigenetic event in mammary carcinoma, however, have never been emphasized until now. Robust and frequent methylation of homeobox genes is not restricted to breast cancer, and occurs at significant frequencies (~10% to 20% of all methylated genes) in early-stage lung carcinoma [28,35] – suggesting a common epigenetic pathway involving the homeobox gene network. Yet, the diverse and nonidentical methylation spectra exhibited by DCIS and stage I lung cancer at homeobox gene-associated CpG islands cautions against the existence of a common epigenetic phenotype among different tumor types. This is not surprising since the pattern and function of the homeobox gene networks are exclusive for a particular tissue [44] and no specific expression and/or CpG-island methylation signatures across tumors have so far been reported.

We cannot deduce why homeobox genes become preferential targets of aberrant CpG methylation during breast tumorigenesis and whether this extensive methylation can shift their finely tuned homeostasis, thus triggering tumorigenesis, or is merely associated with the neoplastic event. The widespread and recurrent nature of this phenomenon, however, seems to suggest that a common mechanistic pathway may exist in cancer cells, which promotes *de novo *methylation of these targets at the onset of tumor development.

Recent data have unraveled the role of Polycomb repressor complexes in targeting and modulating homeobox genes. At least six independent genome-wide studies have identified several common Polycomb targets in vertebrates and flies, most of which are represented by homeobox genes and other developmental transcription factors [45]. Interestingly, 43 out of the 81 annotated genes identified in the present study (~53%) and found to be hypermethylated in early-stage breast cancer overlap with known Polycomb targets, strongly supporting the PcG link [46]. Moreover, most of these DCIS-specific methylated CpG islands are embedded in regions other than promoters, consistent with the finding that the Polycomb repressive complex 2 subunit SUZ12 is distributed across large domains of developmental genes spanning from the promoter up to 2 to 35 kb into the gene [46]. SUZ12 is required for the histone methyltransferase activity and silencing function of the EED–EZH2 complex and is upregulated in different tumors, including breast tumors [47]. EZH2, another key Polycomb repressor complex 2 component, undergoes gene amplification in several tumor types [48] and is overexpressed in prostate cancer and breast cancer [49,50]. EZH2 physically interacts with all three DNA methyltransferases in mammalian cells, and has been suggested to play a crucial role in regulating *de novo *DNA methylation and its maintenance at target sequences [51].

Further support of this mechanistic connection between Polycomb silencing and tumor-associated DNA methylation comes from recent studies linking Polycomb occupancy of genes in noncancerous cells and tissues (including embryonic stem cells) with cancer-associated hypermethylation events [28,35,52-55]. Paradoxically, however, several homeobox genes are upregulated rather than downregulated in breast cancer and other tumor types, suggesting that several tiers of regulation, other than DNA methylation, may concur in determining homeobox misregulation. Several genome-wide PcG profiling studies have reported that 10% to 20% of the identified PcG targets are transcriptionally active [46,56,57]. Bracken and colleagues have suggested that, in undifferentiated cells, PcG complexes have the potential to target genes poised for silencing as well as target genes predisposed to activation [57]. The transition between alternative modes of PcG regulation may require additional signals upon differentiation (and likewise during tumorigenesis), which may include recruitment of additional transcriptional activators and/or competition with PcG antagonists, the tritorax group (trxG) proteins. These signals may all have a counteracting effect to the PcG-mediated gene repression [57].

In a similar scenario, it is conceivable that many of the homeobox gene-associated CpG islands that become methylated in DCIS might have already switched off their active transcriptional state in the normal breast epithelium or its progenitor cells. If that were the case, a hypothesis linking DNA methylation of homeobox gene CpG islands mechanistically to tumorigenesis would not be sustainable. Unfortunately, no RNA samples from DCIS were available to test the relationship between DNA methylation and gene expression.

## Conclusions

Our results strongly suggest that homeobox genes and other developmental transcription factors become preferential targets of *de novo *methylation in DCIS. Aberrant methylation of these master regulators may play a crucial role in the insurgence and/or progression of breast cancer.

## Abbreviations

bp: base pairs; BSA: bovine serum albumin; COBRA: combined bisulfite restriction analysis; DCIS: ductal carcinoma *in situ*; H&E: hematoxylin and eosin; MIRA: methylated-CpG island recovery assay; PCR: polymerase chain reaction.

## Competing interests

The authors declare that they have no competing interests.

## Authors' contributions

ST designed the study, performed the experiments, interpreted the data, and wrote the manuscript. DLK performed the COBRA analysis. XW carried out the statistical analysis of the microarray data. YY contributed with the DCIS specimens and supervised the histological analysis. GPP designed the study, interpreted the data, and wrote the manuscript.
